# Towards Contactless Silent Speech Recognition Based on Detection of Active and Visible Articulators Using IR-UWB Radar

**DOI:** 10.3390/s16111812

**Published:** 2016-10-29

**Authors:** Young Hoon Shin, Jiwon Seo

**Affiliations:** 1 School of Integrated Technology, College of Engineering, Yonsei University, 85 Songdogwahak-ro, Yeonsu-gu, Incheon 21983, Korea; yh.s@yonsei.ac.kr; 2 Yonsei Institute of Convergence Technology, Yonsei University, 85 Songdogwahak-ro, Yeonsu-gu, Incheon 21983, Korea

**Keywords:** IR-UWB radar, contactless silent speech recognition, articulators’ detection

## Abstract

People with hearing or speaking disabilities are deprived of the benefits of conventional speech recognition technology because it is based on acoustic signals. Recent research has focused on silent speech recognition systems that are based on the motions of a speaker’s vocal tract and articulators. Because most silent speech recognition systems use contact sensors that are very inconvenient to users or optical systems that are susceptible to environmental interference, a contactless and robust solution is hence required. Toward this objective, this paper presents a series of signal processing algorithms for a contactless silent speech recognition system using an impulse radio ultra-wide band (IR-UWB) radar. The IR-UWB radar is used to remotely and wirelessly detect motions of the lips and jaw. In order to extract the necessary features of lip and jaw motions from the received radar signals, we propose a feature extraction algorithm. The proposed algorithm noticeably improved speech recognition performance compared to the existing algorithm during our word recognition test with five speakers. We also propose a speech activity detection algorithm to automatically select speech segments from continuous input signals. Thus, speech recognition processing is performed only when speech segments are detected. Our testbed consists of commercial off-the-shelf radar products, and the proposed algorithms are readily applicable without designing specialized radar hardware for silent speech processing.

## 1. Introduction

Automatic speech recognition (ASR) technology has been in use since the mid-20th century and has gradually been applied in diverse fields. In its early days, ASR technology was used to perform simple tasks in applications such as automatic typewriters, automatic call center services, and computer interfaces [1]. With the improvement of its recognition performance, the scope of ASR applications has significantly expanded. However, despite ASR’s usability and convenience, the technology remains limited in terms of stability, accessibility, and security.

Because audio detection is vulnerable to external sound, stable speech recognition is not guaranteed in noisy environments. In addition, from an accessibility perspective, ASR technology is not available to all people; for example, people with speech-related disorders cannot use this technology, even though they can move their articulators. In terms of security, there exists a risk that the user’s speech content can be accessible to other people in the immediate vicinity. For these reasons, several researchers have focused on the novel technology of silent speech recognition.

Silent speech recognition is a speech recognition method that is usable even when an audible acoustic signal is unavailable. In addition to current speech recognition applications, silent speech recognition can also be applied to communications involving people with speech disorders, in noisy environments, and when greater security is required [2]. To enable speech recognition without sound, a variety of sensors have been utilized, such as electromyography (EMG) [3,4,5], electromagnetic articulography (EMA) [6,7,8,9], non-audible murmur (NAM) microphones [10,11,12], ultrasound transducers with video cameras [13,14,15,16], and ultrasound Doppler sensors [17,18].

In [3,4,5], the authors used EMG sensors to capture electrical stimuli from the facial muscles and recorded them for use in speech recognition systems. The advantage of this method is that it is not significantly affected by environmental interference because the sensor is attached directly to the user’s facial muscles. In [6,7,8,9], the EMA device used wired sensor coils that are attached to the surface of the articulators (e.g., the tongue and lips) while the speaker is seated with his/her head within an established magnetic field. The sensor coils were sufficiently small that they could be implanted in the user’s mouth; however, the sensor coils in the mouth were an inconvenience to the users and an established magnetic field is required to utilize this method. In [10,11,12], the authors focused on NAM sounds, which are low-amplitude sounds generated by the resonance of laryngeal airflow in the vocal tract and are not easily overheard by nearby listeners. A speech signal was detected using a high-sensitivity contact microphone attached to the skin; this microphone can detect tissue vibrations during speech and is insensitive to environmental noise. Nonetheless, although these contact sensor-based methods help acquire the positions of articulators or the shape of the vocal tract, they are difficult to use in many practical situations because of their inconvenience.

An alternative method of silent speech recognition combines an ultrasound transducer and a vision sensor to form a contactless silent speech interface [13,14,15,16]. This system uses the movement of the vocal tract as measured by an ultrasound transducer together with a sequence of optical images of the lips. Although this system utilizes contactless sensors, which are much more convenient for users than the previously proposed contact sensors, the vision sensor can often be affected by environmental interference. Moreover, it is difficult to miniaturize the sensor module because this system requires two different sensors to detect the movements of both the lips and vocal tract simultaneously. Ultrasonic Doppler sensing for a silent speech interface was proposed in [17,18]. The method’s potential was demonstrated, but its accuracy on a digit recognition task was only 33% [17].

In contrast, we propose the application of impulse radio ultra-wide band (IR-UWB) radar as a sensor to implement a contactless silent speech recognition system. IR-UWB radar uses electromagnetic waves in an ultra-wide range of radar frequencies and has been applied to diverse applications such as obstacle detection [19], through-the-wall detection [20,21,22], estimation of respiration rates [23,24,25], and object classification [26,27]. IR-UWB radar offers extraordinary resolution and detection precision because its pulses are of short duration. Furthermore, it consumes little power, can be miniaturized, and offers robustness against environmental interference. 

The advantages of using IR-UWB radar in a silent speech recognition system are as follows. First, it uses a contactless sensor that guarantees convenience of users. Second, it is robust against environmental interference such as external sound and light. Third, it has sufficient resolution to detect the position and shape of articulators. Finally, because the size of the IR-UWB radar module has decreased over the years, it can be contained in handheld devices such as tablet PCs and smartphones.

In 2009, Eid and Wallace [28] presented speech sensing using a UWB radar system. The authors developed a UWB antenna for speech sensing and measured the complex reflection coefficients of the signals reflected by vocal tracks with a frequency sweep from 500 MHz to 10 GHz. The UWB antenna was placed within 1–2 cm of the human mouth, and a proof-of-concept experiment with ten words was performed using a simple template matching algorithm based on the delta response of the complex reflection coefficients. To our knowledge, however, this idea has not been further developed since this first publication. Lin et al. [29] developed a Doppler radar system to detect vocal vibration signals. They focused on the speech radar signal acquisition hardware and presented acquired speech radar signals for vowels and words. Although the ability to acquire speech signals using their hardware was demonstrated, speech recognition using the acquired radar signals was not performed. More recently, Chen et al. [30] proposed a method for speech acquisition using a millimeter wave radar in 2015. Their signal processing algorithm enhanced the time-domain waveforms and the spectrograms of the measured radar data, but speech recognition based on the enhanced waveforms and spectrograms was not performed.

In this paper, we propose a combination of signal processing methods to implement a contactless silent speech recognition system based on IR-UWB radar. The foundation for this concept is the observation that when two people cannot communicate using audible sound, the listener focuses on the position and shape of the speaker’s active and visible articulators. Because it is difficult to extract the shape of an entire vocal tract, we focus mainly on active and visible articulators such as the lips and jaw, even though other organs can also be observed by the IR-UWB radar. By observing the reflected radar signal, we found certain features that are related to the position and shape of the articulators. Thus, we developed an algorithm to detect those features from the targets of interest (i.e., the lips and jaw). In addition, we also present a method to detect silent speech activity so that only speech segments from continuous input signals can be automatically stored for the speech recognition processing.

This paper makes the following contributions. First, unlike the previous efforts to design special radar hardware to extract speech-related signals, we use commercial off-the-shelf (COTS) radar products and propose signal processing algorithms that are readily implemented using the COTS hardware. Second, using the IR-UWB radar data, our feature extraction algorithm noticeably improves speech recognition performance compared to the existing algorithm during our simple vocabulary test with five speakers. Third, we propose a speech activity detection algorithm that can automatically select the speech segments from continuous input signals. This algorithm enhances the usability of our system. 

The remainder of this paper is organized as follows: in the next section, we describe the IR-UWB radar signal processing algorithms for implementing the contactless silent speech recognition system. The algorithms include a pre-processing algorithm, automatic silent speech activity detection algorithm, and template matching algorithm for speech recognition. Section 3 evaluates the performance of the proposed system based on experimental results, Section 4 discusses the results, and Section 5 presents the conclusions.

## 2. Method

### 2.1. Overview

**Figure 1:**
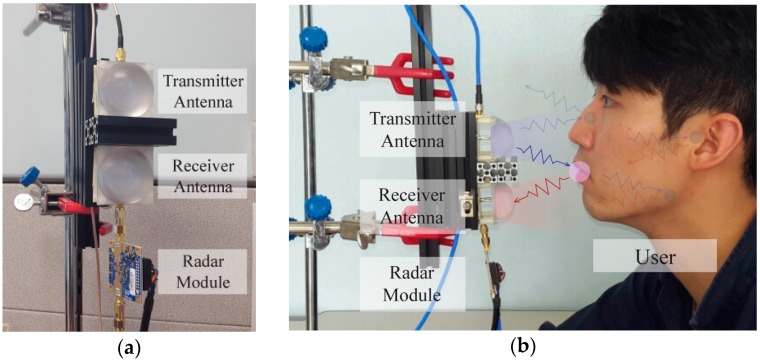
IR-UWB-radar-based silent speech recognition testbed: (**a**) Font view; (**b**) Side view with a user. The transmitted signal is reflected by multiple points on and inside the face. IR-UWB radar signals can penetrate the skin.

Figure 1 shows our testbed for IR-UWB-radar-based silent speech recognition using COTS radar products (detailed specifications of the COTS products are given in Section 3.1). IR-UWB radar signals transmitted from the transmitter antenna are reflected by a speaker’s face. Once the signals are received by the receiver antenna, unwanted reflected signals from the surrounding environment, which is called clutter, need to be reduced. After the clutter reduction, our system detects and extracts certain features about the targets of interest (i.e., the lips and jaw) from the reflected signals. Because the signals are also reflected by unwanted points around the targets and even inside the face, target feature extraction (also called target detection) from the received signals is not a trivial task. Our target detection algorithm extracts the necessary features regarding the position and shape of the lips and jaw.

**Figure 2:**
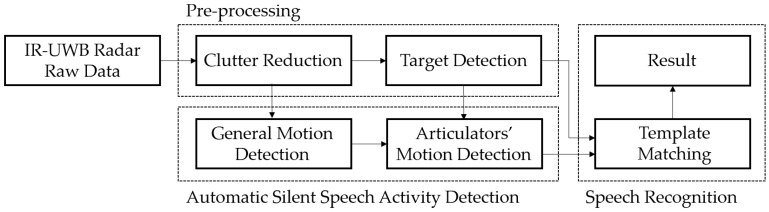
Block diagram of the signal processing flow of the proposed system.

Based on the obtained features, silently spoken words are recognized by a template matching algorithm. This speech recognition processing needs to be performed only when the user moves his/her articulators. Thus, we propose a method to automatically detect silent speech activity. This method consists of two steps. First, the general movement of the user is detected. Once a general motion is detected, the algorithm checks whether the motion is due to the articulators’ movement. When the motion is confirmed as an articulator motion, the speech recognition processing is initiated. The signal processing flow of our silent speech recognition system is illustrated in Figure 2.

### 2.2. Pre-Processing Algorithm

#### 2.2.1. IR-UWB Radar Waveform Model

The proposed method uses an IR-UWB radar equipped with two antennas: one for the transmitter and the other for the receiver. When the electromagnetic wave emitted from the transmitter is scattered by the targets (e.g., the lips and jaw), the receiver antenna receives the reflected wave. From this received signal, we can estimate the position and shape of the targets. After transmitting a single pulse, the radar is expected to receive multiple reflected and distorted pulses. Among these reflected pulses, certain pulses are reflected from the targets, but others, which are normally called clutter, are unwanted signals reflected from the surrounding environment. Thus, it is important to remove clutter in the received signal to analyze the targets of interest correctly. 

There are two time scales for pulse radar, called “fast-time” and “slow-time” [31,32]. The fast-time τ is related to the sampling period of the received signal after transmitting each pulse. The sampled values of the received signal can be stored in a row of a two-dimensional matrix. The slow-time t is related to the interval between the transmitted pulses. Each pulse is transmitted periodically after a pulse repletion interval. After sampling the first received signal corresponding to the first transmitted pulse, the first row of the two-dimensional matrix R is filled. After sampling the second received signal corresponding to the second transmitted pulse, the second row of the matrix is filled, and so forth.

The received analog signal r(t,τ) can be represented by the following equation [25]:

(1) $$r(t,\tau )=\sum_{i}A_{i}p(\tau -\tau_{i})$$

where *p*(*τ*) is the normalized received pulse, *A_i_* is the signal amplitude of the *i*-th received pulse, and *τ_i_* represents the time of arrival of the *i*-th received pulse. The pulses reflected from the targets of interest and the unwanted pulses reflected from the environment (i.e., clutter) are not readily separated in Equation (1). Both of them are received pulses and thus denoted by *A_i_p*(*τ* − *τ_i_*). The received analog signal is sampled periodically in slow-time *t* = *mT_s_* (*m* = 1,2,…,*M*) and fast-time *τ* = *nT_f_* (*n* = 1,2,…,*N*), and the sampled values are stored in matrix *R*:

(2) $$R[m,n]=r(mT_{s},nT_{f})$$

**Figure 3:**
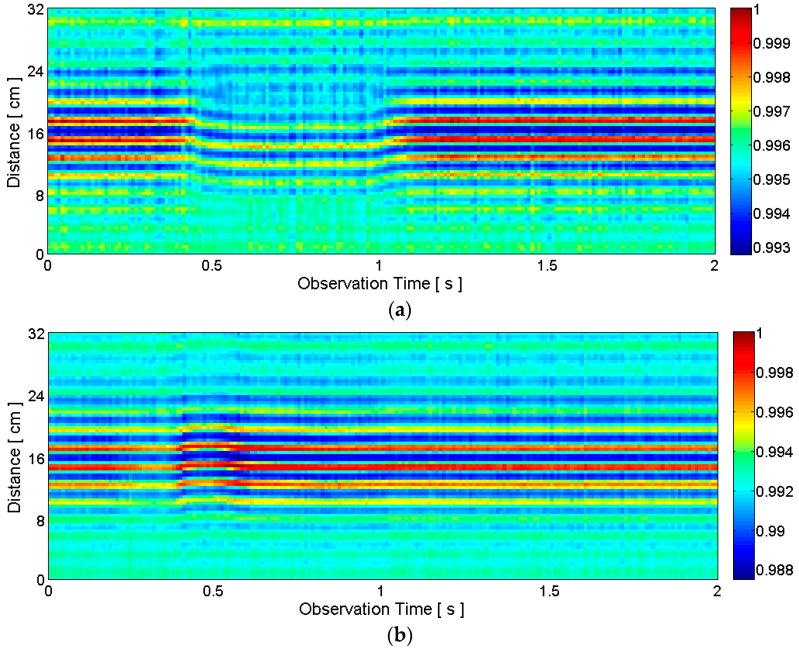
Examples of raw received radar signals corresponding to: (**a**) silent pronunciation of the word “two”; (**b**) silent pronunciation of the word “five”. The approximate beginning time (about 0.4 s) and end time (about 1.1 s) of the pronunciation of “two” is clearly visible, but they are not very clear for “five” in this raw data.

Matrix *R*, which contains the raw received radar signal, can be visualized as in Figure 3. Because the observation time, which is the slow-time, is on the *x*-axis, and the distance, which is related to the fast-time, is on the *y*-axis, the visualization in Figure 3 matches *R^T^*.

The raw radar data for the silent pronunciation of the word “two” is visualized in Figure 3a. Because the lips move closer to the radar when pronouncing “two”, the distances measured during the pronunciation decrease. This phenomenon is clearly visible between about 0.4 s and 1.1 s. The downward curves indicate decreased distances to the objects. Note that there are several horizontal curves with high signal amplitudes in Figure 3a. This is because the transmitted pulse is reflected by multiple points on and inside the face even though we aim at the lips and jaw. Another observation is the decrease in signal amplitude, indicated by yellow and green colors during the pronunciation. This happens because the surface area of the lips and jaw at the closest distance to the radar is now almost determined by the surface area of the lips alone. The jaw has little influence on the surface area in this case because the lips have moved forward and are now closer to the radar compared to the jaw. This decreased surface area at the closest distance caused by the movement of the lips causes the signal amplitude of the received signal to decrease. Of course, the actual physical phenomena behind this amplitude change are complex, but we can understand that the amplitude change is at least related to the change in shape of the articulators.

Figure 3b visualizes the case of “five” and the result is very different from the case of “two”. Now the lips stay at the almost same distance and the jaw drops downward. Because this jaw movement is perpendicular to the direction toward the radar, the distance measurements show little variations. Now the surface area of the lips and jaw at the closest distance to the radar slightly increases during the pronunciation because of the dropped jaw. Thus, the signal amplitude does not decrease as in the case of “two.” In fact, the signal amplitude slightly increases during the pronunciation even though it is not clearly visible given the scale of Figure 3b.

#### 2.2.2. Clutter Reduction

In this study, clutter is defined as signals produced by reflections from multiple background points, which are not the points on the targets of interest, as well as interference caused by the coupling between the transmitter and receiver antennas. We apply an existing clutter reduction algorithm based on signal averaging [33]. Let *r*[*n*] denote a row of matrix *R*. The length of vector *r*[*n*] is *N*, which is the number of “fast-time” epochs used in the analysis. Without placing any objects in front of the radar, we can obtain a raw signal matrix *B* that represents the reflected radar signals from the background points. Clutter-reduced signal *y*[*n*] can then be calculated as follows:

(3) $$y[n]=r[n]-\frac{1}{M_{B}}\sum_{m=1}^{M_{B}}B[m,n]$$

where *M_B_* is the number of “slow-time” epochs over which the signals are averaged for clutter reduction, and *M_B_* = 100 is used in our experiments.

**Figure 4:**
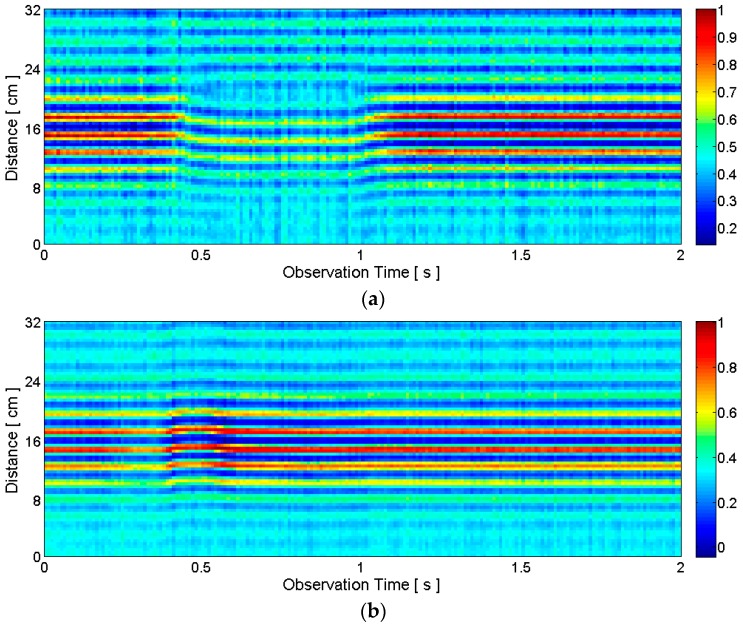
Examples of clutter-reduced signals corresponding to: (**a**) silent pronunciation of the word “two”; (**b**) silent pronunciation of the word “five”. The raw radar data is the same as the data in Figure 3.

In the following discussion, *y*(*τ*) is the received analog signal for a single radar scan (i.e., for a single transmitted pulse) after the clutter reduction, and *y*[*n*] is the sampled version of *y*(*τ*). After a series of radar scans, we can construct a clutter-reduced signal matrix *Y*[*m,n*] whose rows are the one-dimensional clutter-reduced signal vectors. Figure 4 visualizes some examples of *Y*[*m,n*] matrices (more precisely, these figures correspond with *Y^T^*).

#### 2.2.3. Target Detection

Our IR-UWB-radar-based silent speech recognition system requires an adequate method for detecting the position and shape information of the targets of interest (i.e., the lips and jaw). As shown in Figure 3, the distance measurement is sensitive to the position of the lips, and the signal amplitude measurement is sensitive to the surface area of the lips and jaw at the closest distance to the radar (i.e., sensitive to the shape of the lips and jaw). Thus, the distance and signal amplitude measurements contain the necessary position and shape information of the lips and jaw. Target detection in this paper means the detection and extraction of the desired features of the targets of interest from the received signals. Target detection is not trivial because the transmitted pulse is reflected by many undesired points around the targets of interest, which inevitably contaminates the desired information.

A number of algorithms for target detection using an IR-UWB radar have been proposed, such as the interperiod-correlation processing (IPCP) detector [34], constant false alarm rate (CFAR) detector [35], discrete-cosine-transform (DCT)-based approach [36], and CLEAN detection algorithm [37,38]. CLEAN [37,38] is a high-resolution deconvolution algorithm that was first used to enhance radio-astronomical imaging of the sky [37]. It has also been widely used as a target detection method in conjunction with IR-UWB radar [38]. However, the CLEAN algorithm has limits when directly applied to our task. Because the distances between articulators are shorter than the length of a single transmitted pulse, the received pulses from different articulators can overlap, causing target detection error when the template pulse is subtracted from the distorted reflected signal using the conventional CLEAN algorithm. Our short-template-based CLEAN algorithm, which is a target detection method modified specifically for our task, overcomes this limitation.

The conventional CLEAN algorithm can be described as follows [38].

(1) Obtain the sampled values of a transmitting pulse that is not reflected by any object as CLEAN template *v*[*n*]. This template can be obtained by directly connecting the transmitter and receiver part of the radar by a cable.
(2) Initialize the dirty map, clean map, user-defined stop threshold, and iteration counter as *d*_0_[*n*] = *y*[*n*], *c*_0_[*n*] = 0, *T_stop_* and *i* = 1, respectively.
(3) Compute the normalized cross-correlation between the dirty map and CLEAN template:
  $$s[n]=d_{i-1}[n]⊙v[n].$$
(4) Find the maximum correlation value and its argument:
  $$a_{i}=\max s[n],n_{i}=arg\max s[n].$$
(5) If *a_i_* < *T_stop_*, stop. Go to Step 9.
(6) Clean the dirty map by subtracting the template modified by *a_i_* and *n_i_* from it: *d_i_*[*n*] = *d_i_*_-1_[*n*] − *a_i_v*[*n* − *n_i_*].
(7) Update the clean map: *c_i_*[*n*] = *c_i_*_-1_[*n*] + *a_i_δ*[*n* − *n_i_*], where *δ* is the Dirac delta function.
(8) Increase the iteration counter *i* = *i* + 1, and return to Step 3.
(9) When the iteration stops, store clean map *c_i_*[*n*].

**Figure 5:**
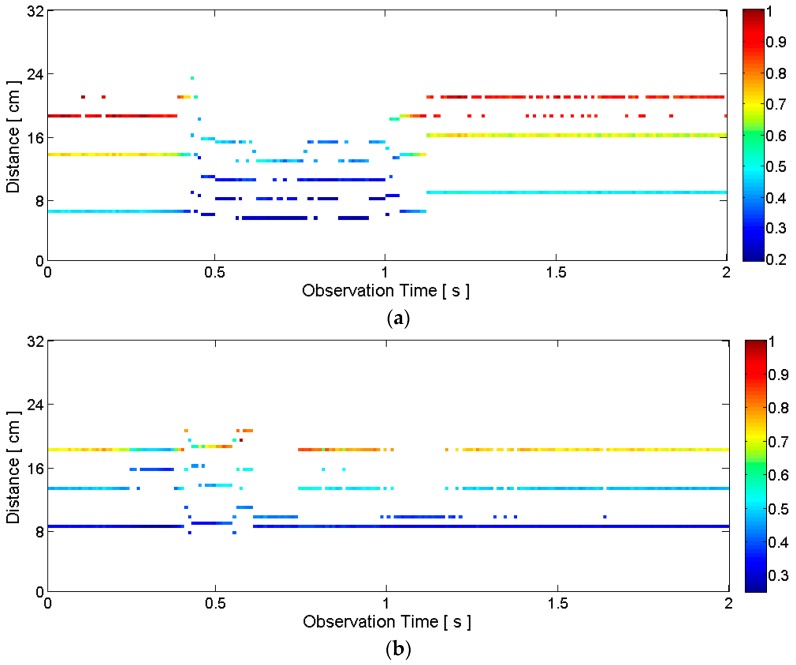
Example clean maps obtained by the conventional CLEAN algorithm corresponding to: (**a**) silent pronunciation of the word “two”; (**b**) silent pronunciation of the word “five”. The raw radar data is the same as the data in Figure 3 and Figure 4.

Alternatively, instead of the delta function, the CLEAN template modified by *a_i_* and *n_i_* can be added to the clean map in Step 7 (i.e., *c_i_*_-1_[*n*] + *a_i_v*[*n* − *n_i_*]). For each radar scan, a clean map in the form of a one-dimensional vector *c_i_*[*n*] is obtained. After a series of radar scans, we can construct a two-dimensional clean map matrix *C*[*m,n*] whose rows are the one-dimensional clean map vectors. This clean map matrix is visualized in Figure 5 with the same raw data set as in Figure 3 and Figure 4. Comparing Figure 5 to Figure 4, only the distances and correlation amplitudes of desired pulses, which are represented by *a_i_δ*[*n* − *n_i_*] remain. A lower *T_stop_* value leaves more data points in the clean map, and a higher *T_stop_* leaves fewer data points. We can select an appropriate threshold for a given application. (Figure 5 is the case of *T_stop_* = 2 with our testbed.)

The basic idea of the conventional CLEAN algorithm is to extract the desired pulses, which are represented by high cross-correlation with the transmitted (template) pulse *v*[*n*], from the received signal, which contains both desired and undesired pulses. Thus, the algorithm assumes that the received signal is a summation of delayed and scaled versions of the transmitted pulse. However, if the target has a complex shape, the transmitted pulse can be distorted when reflected from the target, resulting in a mismatch between the template pulse *v*[*n*] and the reflected pulses. Therefore, a cumulative error occurs when subtracting the template pulse from the received signal in Step 6. Several methods have been proposed to solve this problem, such as the multi-template deconvolution method [38] and zeroing method [39]. However, in our case, it is difficult to obtain accurate multi-templates from different types of active articulators, and the zeroing method can potentially eliminate desired pulses located near the pulse with the maximum cross-correlation. Therefore, a different approach is needed for our silent speech recognition application.

By careful observations, we realized that the overlaps of the distorted pulses in the received signal degrade the performance of the system. We also observed that the reflected signal from the nearest target experiences the smallest amount of overlap. In order to detect the nearest target robustly, we propose to use a short template *v_s_*[*n*]. Our short template consists of 25% of the front part of the original template *v*[*n*] and the remaining 75% consists of zeros. Thus, if the second reflected pulse does not overlap 75% of the first reflected pulse in the received signal, this short-template-based cross-correlation can detect the first reflected pulse. Unlike the conventional CLEAN algorithm, which contains iterations, our short-template-based CLEAN algorithm stops after detecting the first reflected pulse because other pulses may be contaminated by overlapping pulses. The first reflected pulse from the nearest target likely contains the position and shape information of the lips and jaw.

The proposed short-template-based CLEAN algorithm is described as follows. This algorithm is simple and provides noticeable performance benefit for our application over the conventional CLEAN, as discussed in Section 3 with experimental data.

(1) Obtain the conventional CLEAN template; then generate short template *v_s_*[*n*].
(2) Compute the normalized cross-correlation between *y*[*n*] and the short template:
  $$s[n]=y[n]⊙v_{s}[n].$$
(3) Find the maximum correlation value and its argument:
  $$a_{\max}=\max s[n],n_{\max}=arg\max s[n].$$
(4) Store the clean map: *c*[*n*] = *a*_max_ *δ* [*n* − *n*_max_].

**Figure 6:**
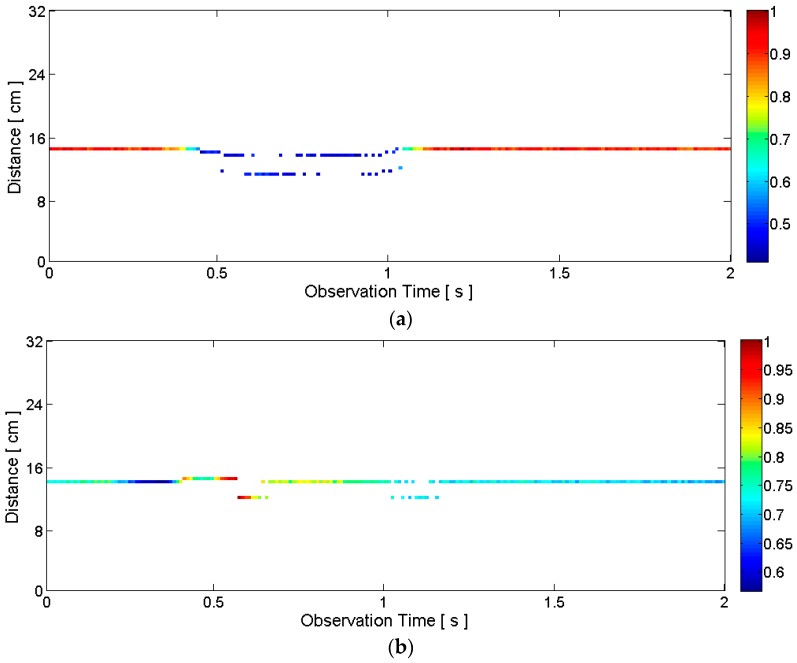
Example clean maps obtained by the short-template-based CLEAN algorithm: (**a**) silent pronunciation of the word “two”; (**b**) silent pronunciation of the word “five”. The raw radar data is the same as the data in Figure 3, Figure 4 and Figure 5. Unlike the raw data in Figure 3b, the approximate beginning time (about 0.3 s) and end time (about 1.1 s) of the pronunciation of “five” is now clearly visible.

Following these steps for each radar scan, a clean map matrix is obtained. Figure 6 provides example clean maps obtained by the short-template-based CLEAN. Unlike Figure 5, there is only one data point per each observation epoch in Figure 6. Each data point contains the distance and correlation amplitude information. This distance and correlation amplitude information is used as the features for speech recognition. The distance contains the position information of the lips, and the correlation amplitude is related to the signal amplitude that contains the shape information of the lips and jaw. The approximate articulation time of “five”, which was not evident in the raw data in Figure 3b, is now clearly visible (i.e., between about 0.3 s and 1.1 s) in Figure 6b.

### 2.3. Automatic Silent Speech Activity Detection 

Speech activity detection, or endpoint detection, has been actively studied for ASR [40] but has not been studied for IR-UWB-radar-based silent speech recognition. Because a speech activity detection algorithm can automatically select the speech segments from continuous input signals, it is useful for accumulating large amounts of training data. It can also reduce the computational load of speech recognition processing by removing non-speech segments. As a result, speech recognition is performed only when a speech segment is detected. Our silent speech activity detection algorithm consists of two steps: general motion detection and articulators’ motion detection. First, any movement of the speaker is detected based on the radar data in the general motion detection step. We then check whether the detected motion is due to articulator motion.

#### 2.3.1. General Motion Detection

When the objects in front of the radar move, the received signal amplitude changes. Thus, the statistical variance of the signal amplitude change at a certain observation time can be used as a decision parameter for motion detection. The normalized signal amplitude difference at a certain distance *x*[*n*] between the observation epochs *m* and *m* − 1 is expressed as follows:

(4) $$x[n]=\frac{\left|Y[m,n]-Y[m-1,n]\right|}{\sum_{k=1}^{N}\left|Y[m,k]-Y[m-1,k]\right|}$$

where *Y* is the clutter-reduced signal matrix. We then calculate the variance of *x*[*n*] at observation epoch *m*:

(5) $$\mu_{m}=\frac{1}{N}\sum_{n=1}^{N}x[n]=\frac{1}{N}$$

(6) $$\sigma_{m}^{2}=\frac{1}{N}\sum_{n=1}^{N}{\left(x[n]-\mu_{m}\right)}^{2}=\frac{1}{N}\sum_{n=1}^{N}{\left(x[n]-\frac{1}{N}\right)}^{2}$$

Because this variance is noisy, we apply the exponential moving average filter with *α* = 0.1:

(7) $${\tilde{\sigma }}_{m}^{2}=\alpha \sigma_{m}^{2}+(1-\alpha ){\tilde{\sigma }}_{m-1}^{2}$$

**Figure 7:**
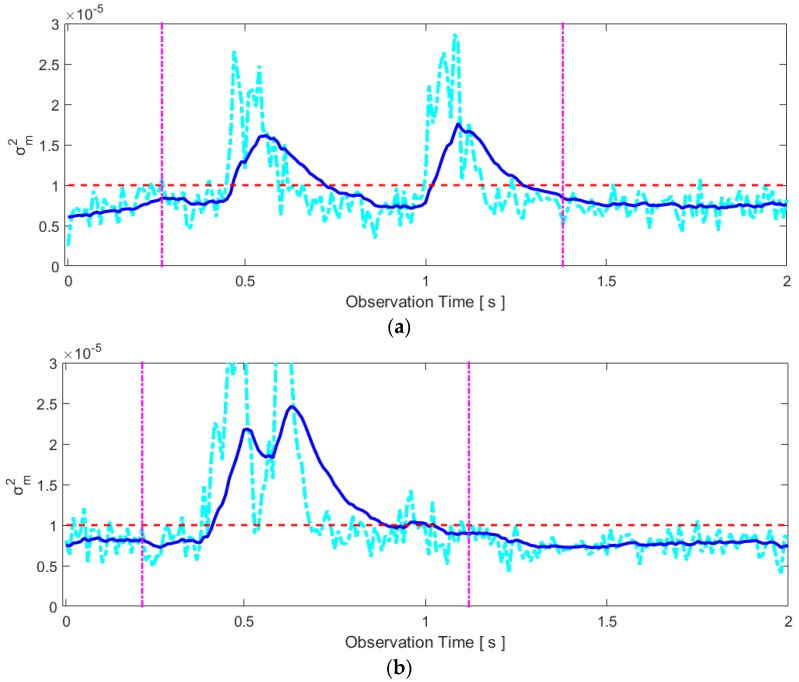
Examples of the variance of normalized signal amplitude (raw variance data and smoothed data): (**a**) silent pronunciation of the word “two”; (**b**) silent pronunciation of the word “five”. The raw radar data is the same as the data in Figure 3, Figure 4, Figure 5 and Figure 6. The values above the threshold (horizontal dashed line) indicate the general motion of the speaker. The data between the vertical lines are stored for further processing.

The noisy $\sigma_{m}^{2}$ and smoothed ${\tilde{\sigma }}_{m}^{2}$ are shown in Figure 7 for the same raw data set used in Figure 3, Figure 4, Figure 5 and Figure 6. In our experimental setup, the number of utilized samples per each radar scan is *N* = 256. In this case, we set the threshold for detecting motion to 1 × 10^−5^. Obviously, there is little variation in the variance of a normalized signal amplitude when the speaker does not move. Thus, the threshold to detect the user’s movement was selected based on no-movement data and the same threshold was applied to all speakers and words during our experiment.

When “two” is pronounced, the lips move forward, pause, and move backward. These motions are captured by the two bumps above the threshold in Figure 7a. If the smoothed variance is below the threshold for more than 0.4 s, we decide the silent pronunciation of a word is finished. We may then automatically store the data between 0.47 s and 1.28 s, in the case of Figure 7a, by applying the threshold to the smoothed variance. Note, however, that the beginning and end times of the motion detected by this method are slightly lagged because of the average filter. In order not to lose any data during the actual articulation, we apply a 0.2 s margin to the beginning time and a 0.1 s margin to the end time. Thus, the data between 0.27 s and 1.38 s are automatically stored for further processing in this case. In the case of Figure 7b, the intersections with the threshold occur at 0.42 s and 1.02 s. Thus, the data between 0.22 s and 1.12 s are stored.

#### 2.3.2. Articulators’ Motion Detection

**Figure 8:**
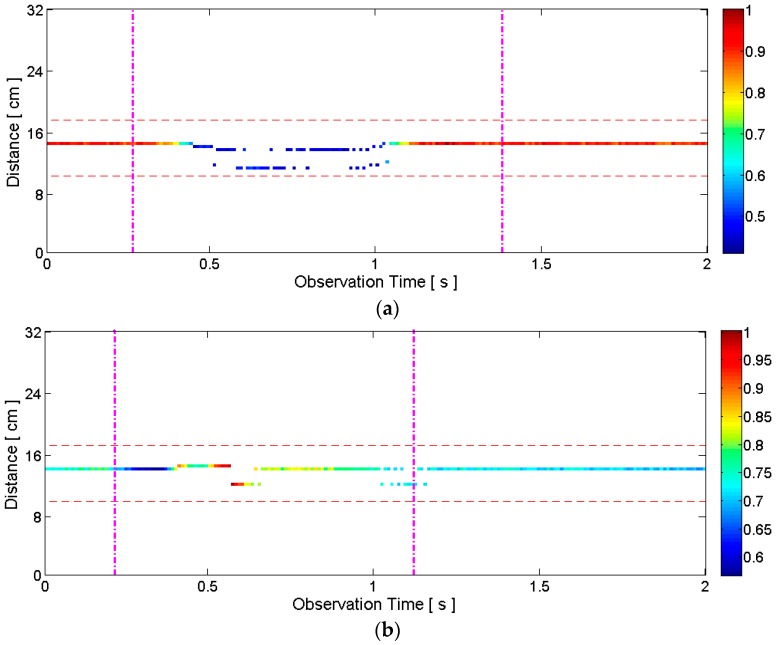
Example clean maps with the distance thresholds (horizontal dashed lines): (**a**) silent pronunciation of the word “two”; (**b**) silent pronunciation of the word “five”. The raw radar data is the same as the data in Figure 3, Figure 4, Figure 5, Figure 6 and Figure 7. Both data within the distance thresholds represent articulator motion, and thus the data segments between the vertical lines are stored.

The general motion detection algorithm described in the previous subsection detects any motion of the speaker such as head movement. Thus, the data stored after the general motion detection needs to be tested to determine if it really represents an articulation. For this purpose, the distance information in the clean map obtained by short-template-based CLEAN is used. If the distance measurement of the stored data is between −4 cm and 3 cm with respect to the distance measured when there is no motion, we decide the movement is due to articulator motion. These thresholds are selected by assuming that the maximum forward and backward movements of the lips are less than 4 cm and 3 cm, respectively, during articulation. Note that these thresholds include margins and successfully captured all the articulations of the five speakers during our experiment. Figure 8 illustrates the thresholds on top of the data of Figure 6.

If the change in distance was larger than the distance thresholds, the effect of other motions such as head movement was included in the measurement data. Thus, our current algorithm discards the data and does not proceed to the following silent speech recognition processing. However, a very small head movement within the thresholds is not detected by this method and it can degrade the performance of our system. Note also that our system currently ignores the problem of non-speech movements of the articulators. If the measured changes in distance due to a non-speech movement of the articulators were within the distance thresholds, the data was stored for further processing in our current implementation, although this is not desirable. We cannot distinguish speech movements and non-speech movements of the articulators based on the radar data alone. This distinction may be possible at the recognition step.

### 2.4. Template Matching for Speech Recognition

In general, the durations of two articulations are different. Thus, we used the dynamic time warping (DTW) algorithm [41] for speech recognition. Specifically, the multi-dimensional dynamic time warping (MD-DTW) in [42] was applied because the clean map provides two features (i.e., correlation amplitude *a*_max_ and corresponding distance *n*_max_ for short-template-based CLEAN; maximum correlation amplitude *a*_1_ and corresponding distance *n*_1_ for conventional CLEAN). Using clean map matrix *C*[*m,n*] we can construct two-dimensional feature matrix *F*[*i,k*], where *k* = 1,2, by storing two feature values per each observation epoch *i*. Index *i* represents the observation time between the two vertical lines in Figure 8, for example. Obviously, the number of rows of matrix *F* varies in general even though the same word is pronounced repeatedly. The distance matrix between template feature matrix *F_temp_* and test feature matrix *F_test_* is calculated as follows [42]:

(8) $$Dist[i,j]=\sum_{k=1}^{2}\left|F_{temp}[i,k]-F_{test}[j,k]\right|$$

**Figure 9:**
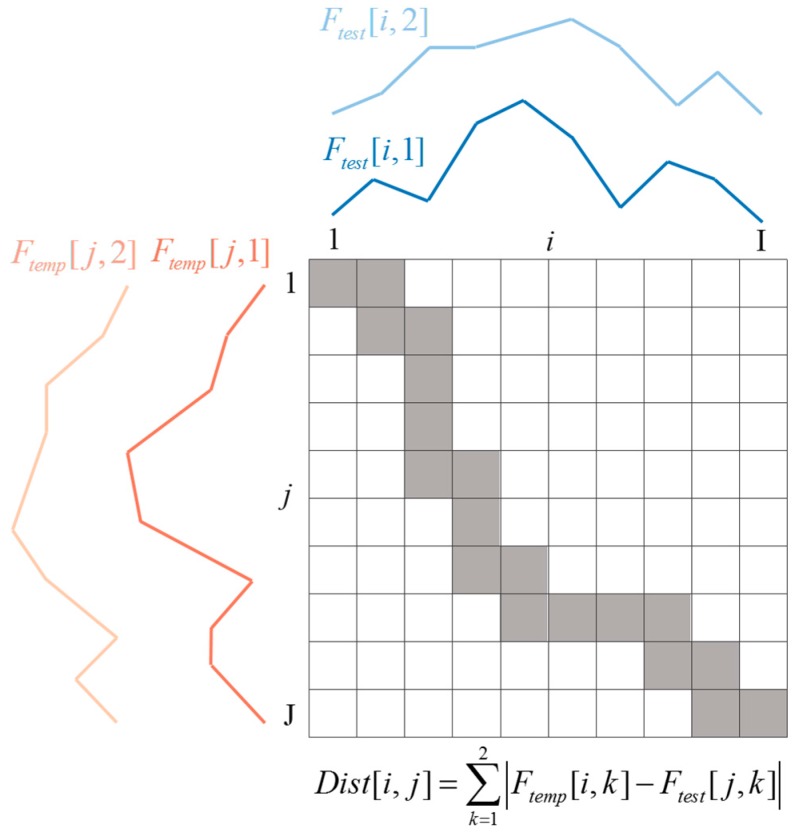
Illustration of the distance matrix and alignment path of the MD-DTW algorithm for two features. Each (*i*, *j*) element of the matrix contains a distance value calculated by Equation (8). The alignment path in gray is the path having the minimal total distance value.

The regular DTW algorithm is then applied using this distance matrix. The distance matrix and alignment path of the MD-DTW algorithm are illustrated in Figure 9.

## 3. Evaluation

### 3.1. Experimental Setup

We preformed several experiments to evaluate the performance of our proposed system. The COTS IR-UWB radar system used in our testbed is a product of Novelda [43] and it is equipped with the NVA6200 CMOS impulse radar chipset with a frequency range from 6 to 10.2 GHz. The radar is connected to two COTS sinuous antennas with a beam range of approximately 40° (vertical) × 35° (horizontal). In order to reduce the antenna coupling, an aluminum profile was placed between the two antennas, as shown in Figure 1.

Five speakers participated the experiments. Their native language is Korean, but they have 13–15 year English education and do not have any problem to correctly pronounce the ten simple English words used in this study. We specifically instructed them to keep their heads stationary during articulations because our current algorithm does not compensate for any small head movement. Data with a large head movement is detected and discarded at the articulators’ motion detection step in Section 2.3.2. The horizontal distance between the radar and a speaker was approximately 10–16 cm during the experiments. Because our algorithm is not sensitive to horizontal distance, this distance can vary for each test. However, the actual usable distance depends on the radar performance. A higher signal power can increase the usable distance, but the size of the system required and its power consumption also increase. A 10–16 cm distance is a reasonable choice considering the distance between a hand-held device and a user’s mouth. The distance resolution of the received radar signal is 4 mm, and we obtained a radar scan every 0.01 s.

### 3.2. Performance of the Proposed Silent Speech Recognition System

First, a simple vowel recognition test using our proposed method was performed. A speaker was asked to silently read the set of five English vowels 20 times in a single session. These 100 samples (5 vowels × 20 repetitions) were used for evaluation. As in [44], the jackknife method was used for cross-validation. From the set of 100 samples, one test sample was selected and compared to the other 99 samples using the MD-DTW method (i.e., a test vowel that can be considered as a newly uttered vowel was compared to 19 repetitions of the target vowel and 20 repetitions of each non-target vowel). The recognition decision was made by the vowel of the closest match. After repeating this comparison 100 times by selecting different test samples, the recognition rates in terms of precision and recall were obtained (Table 1). The F-measure was 0.943 in this experiment. Table 2 contains the average classification result for each spoken vowel. The minimum functionality of the proposed system was shown by this simple test with one speaker.

Second, a word recognition test was then performed with five speakers (5 males, aged 23–28 years) and ten English words from “one” to “zero”. Each speaker silently spoke the words in a single session, but the relative position between a speaker and the radar was not fixed but different for each session. The 1000 samples (10 words × 20 repetitions × 5 speakers) were used for the evaluation. The same jackknife method used in the vowel recognition test was used for cross-validation. The cross-validation was performed for each speaker independently. In order to evaluate the performance of our short-template-based CLEAN algorithm, we processed the same radar data set using two different target detection algorithms: the conventional CLEAN and the short-template-based CLEAN. The other processing algorithms remained the same for fair comparison. Recall that the conventional CLEAN provides multiple data points on the clean map per each observation epoch and we cannot distinguish the data points representing the lips and jaw. In this experiment, we selected the data point with the maximum correlation amplitude at each observation epoch for the recognition processing.

**Figure 10:**
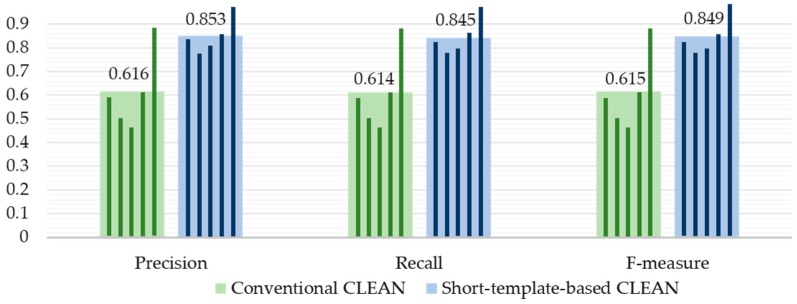
Comparison of precision, recall, and F-measure of word recognition with five speakers. Each narrow bar indicates the result of each speaker, and each wide bar and corresponding number indicates the average value over five speakers.

The precision, recall, and F-measure of the word recognition test using conventional CLEAN and short-template-based CLEAN are given in Figure 10. Table 3 and Table 4 contain the average classification result for each spoken word using conventional CLEAN and short-template-based CLEAN, respectively. This result clearly demonstrates the performance benefits of the proposed method.

This result also shows the limits of the proposed system. The average precision of speaker 2 using short-template-based CLEAN is 0.785 (Figure 10), which is significantly lower than the average precision of speaker 5 which is 0.966. Speaker 5 was one of the authors of this paper and thus very experienced at silently speaking a word without moving his head. Speaker 2 obviously did not have this skill and his head movement reduced the performance of the proposed system (a large head movement is detectable by our articulators’ motion detection algorithm, but a small head movement within the thresholds is undetectable). In addition, note that the degradation in performance between speakers 2 and 5 was more significant when the conventional CLEAN method was used. The precision of 0.894 of Speaker 5 was reduced to 0.505 for Speaker 2 using conventional CLEAN, as shown in Figure 10. Thus, our short-template-based CLEAN method is less sensitive to head movements than conventional CLEAN.

## 4. Discussion

Because a very small-scale COTS IR-UWB radar system has recently become available, its applications have expanded rapidly. The IR-UWB radar system used for our testbed, for example, contains all essential radar subsystems within a 5 mm × 5 mm package [43]. The objective of our study is to verify the feasibility of contactless silent speech recognition using small-scale COTS radar. Previous efforts in the literature mainly focused on a radar hardware design that is optimized for speech recognition. However, we focused on signal processing algorithms that are readily implemented using general small-scale COTS radar. The proposed algorithms demonstrated about a 85% word accuracy for 10 isolated words, which is comparable to the previous silent speech recognition results using contact sensors (e.g., EMA, EMG, and NAM) and contactless sensors (e.g., ultrasound and optical sensors). For example, Wang et al. [45] used EMA and obtained a recognition accuracy of 80%–97% for 25 words. Schultz and Wand [3] presented an EMG-based speech recognition with an accuracy of about 90% for 100 words. Heracleous et al. [46] achieved a 93.5% word accuracy using 24 NAM utterances. Hueber et al. [47] utilized ultrasound and optical sensors and achieved about a 75% accuracy for 2500 words.

The main performance degradation of our proposed system was due to the head movement of the speaker. Thus, an additional algorithm to compensate for head movement needs to be developed to enhance its performance. Further, the current evaluation was only on isolated sounds. The current two features (i.e., distance and correlation amplitude) are not enough to enable phoneme-level recognition, as it is important to extract features from tongue movements for phoneme-level recognition. This is theoretically possible because the IR-UWB radar signals can easily penetrate the skin, but a more sophisticated target detection algorithm should be developed. The visualization of the lips and tongue motion for speech therapy could be an interesting application of IR-UWB radar. The visualization of the lips’ motion is possible using the features obtained by our algorithm, but tongue motion visualization requires further research efforts.

## 5. Conclusions

Silent speech recognition technology is not only useful for aiding speech recognition in noisy environments, but also allows people with hearing or speaking disabilities to benefit from speech-recognition-based applications. Because IR-UWB radar is a contactless sensor and is robust to environmental interferences, it can be considered a desirable sensor for implementing a contactless silent speech recognition system. In this paper, we proposed an IR-UWB-radar-based contactless silent speech recognition system and experimentally demonstrated its performance. We identified appropriate features that can be obtained from radar signals reflected from articulators for use in the proposed algorithms. The full set of signal processing steps is presented. In the experiments performed with five speakers, our system demonstrated an accuracy of about 85% for 10 isolated words.

## Figures and Tables

**Table 1:** Precision and recall of vowel recognition using the proposed method. Five English vowels (a, æ, i, ɔ, u) are tested.

	a	æ	i	ɔ	u	Average
Precision	0.833	1.000	0.950	0.944	1.000	0.946
Recall	1.000	0.950	0.950	0.850	0.950	0.940

**Table 2:** Result of vowel recognition using the proposed method. Five English vowels (a, æ, i, ɔ, u) are tested.

Spoken Vowel	Recognized Vowel (%)				
	a	æ	i	ɔ	u
a	100	0	0	0	0
æ	0	95	5	0	0
i	5	0	95	0	0
ɔ	15	0	0	85	0
u	0	0	0	5	95

**Table 3:** Result of word recognition with five speakers using conventional CLEAN algorithm.

Spoken Word	Recognized Word (%)									
	One	Two	Three	Four	Five	Six	Seven	Eight	Nine	Zero
One	76	7	2	0	4	2	4	4	0	1
Two	5	74	8	3	0	1	2	2	2	3
Three	4	6	56	6	5	5	7	1	8	2
Four	4	2	7	63	3	4	5	3	7	2
Five	2	2	3	5	56	3	12	13	2	2
Six	2	0	7	3	6	52	6	11	10	3
Seven	9	2	7	3	9	4	51	7	4	4
Eight	5	3	2	4	8	5	5	51	5	12
Nine	3	1	5	3	1	2	4	8	73	0
Zero	1	0	6	4	4	4	2	13	4	62

**Table 4:** Result of word recognition with five speakers using the short-template-based CLEAN algorithm.

Spoken Word	Recognized Word (%)									
	One	Two	Three	Four	Five	Six	Seven	Eight	Nine	Zero
One	84	10	0	1	0	2	0	2	0	1
Two	6	90	0	0	1	1	0	0	2	0
Three	1	0	84	0	4	2	0	4	5	0
Four	1	0	2	87	2	1	2	3	2	0
Five	3	0	2	1	82	3	6	1	2	0
Six	1	1	3	0	5	82	2	3	2	1
Seven	5	1	3	1	4	2	78	2	2	2
Eight	0	0	9	2	0	0	2	83	0	4
Nine	1	0	0	2	1	2	3	2	89	0
Zero	3	0	1	0	3	1	2	4	0	86
